# Nutritional Status Assessment in Cirrhotic Patients after Protein Supplementation

**DOI:** 10.5402/2012/690402

**Published:** 2012-12-04

**Authors:** Supanee Putadechakum, Theerawut Klangjareonchai, Arpussanee Soponsaritsuk, Chulaporn Roongpisuthipong

**Affiliations:** ^1^Research Center, Faculty of Medicine, Ramathibodi Hospital, Mahidol University, Bangkok 10400, Thailand; ^2^Department of Medicine, Faculty of Medicine, Ramathibodi Hospital, Mahidol University, Bangkok 10400, Thailand

## Abstract

*Background*. Protein supplementation has been shown to be effective for the treatment of malnourished patients with liver cirrhosis. The parameters used to assess nutritional improvement in cirrhotic patients for such treatment are important. *Objective*. To evaluate the parameters for assessment of nutritional status in patients with liver cirrhosis after protein supplementation. *Material and Method*. A cross-sectional, prospective clinical trial with 22 cirrhotic patients was performed. Data from anthropometry, bioelectrical impedance, subjective global assessment (SGA), and visceral protein were gathered and analyzed to assess nutritional improvement after protein supplementation. *Results*. Twenty-two cirrhotic patients (mean age 52.9 ± 12.8 years; 54.5% male; 63.6% alcoholic cirrhosis; 63.6% Child-Pugh C) were recruited. After protein supplementation, a significant improvement was demonstrated in the SGA class A from 10 patients (45.5%) to 16 (72.7%) and 18 (81.8%) at the 4th and 8th weeks, respectively. Body weight, body mass index, and lean muscle mass were significantly increased from baseline at the 8th week. No significant change in other nutritional parameters was observed. *Conclusions*. The SGA and lean muscle mass were significant parameters in order to assess nutritional status in cirrhotic patients after protein supplementation.

## 1. Introduction

Cirrhotic patients often suffer from malnutrition due to decrease in nutrient consumption or impaired liver metabolism [1, 2]. Cirrhotic patients with malnutrition have been recognized to have greater risk for increased postoperative complications and mortality [3, 4]. A variety of mechanisms are considered to contribute to malnutrition in cirrhosis such as poor food intake, malabsorption, increased intestinal protein loss, decreased protein synthesis, disturbances in substrate utilization, and hypermetabolism. Vegetative protein containing branched-chain amino acid (BCAA) supplementation has been shown to be effective for treatment of malnourished patients with liver cirrhosis [5]. The parameters used to assess nutritional improvement of cirrhotic patients are important for such treatment. There were many suggested parameters to assess the nutritional improvement from previous studies. In this study, six parameters including body weight, body composition, Triceps skinfold thickness (TST), subjective global assessment (SGA), serum albumin, and prealbumin were used and the results were gathered and analyzed to assess nutritional improvement in patients with liver cirrhosis after vegetative protein supplementation.

## 2. Material and Method

### 2.1. Subjects

Twenty-two patients who were diagnosed of having liver cirrhosis based on clinical and histological evidences, or imaging diagnosis, were recruited from Ramathibodi Hospital, Bangkok, Thailand. Clinical evidence of cirrhosis was defined with the presence of portal hypertension and hepatic insufficiency [6]. The severity of liver cirrhosis was graded using Child-Pugh scores [7]. The study excluded patients with hepatocellular carcinoma, poor intestinal absorption, chronic renal disease, acquired immunodeficiency syndrome, recent alcohol drinking, and those who refused to join in this study. Informed consent was obtained from all participants prior to enrollment. The study protocol had been reviewed and approved by the ethical committee on human research of Ramathibodi Hospital, Mahidol University.

### 2.2. Study Design

The study was a cross-sectional, prospective clinical trial. Each patient, among all 22 patients who visited the nutrition clinic, received 20 grams of vegetable protein (soy) supplementation per day add on their regular diet for 8 weeks. The detail nutritional composition of the supplements used in this study is shown in Table 1.

### 2.3. Nutritional Assessment

Nutritional assessment was based on the following: anthropometry, bioelectrical impedance, visceral protein, and subjective global assessment (SGA). All measurements were taken by the same investigator to avoid any interobserver variation at baseline, the 4th week and the 8th week.

### 2.4. Anthropometry

Body weight was measured by Soehnle 7755, a digital weighing scale (Soehnle Professional, Backnang, Germany). Triceps skinfold thickness (TST) was measured by Harpenden skinfold caliper (Inter Reha, Tokyo, Japan).

### 2.5. Bioelectrical Impedance

Body composition was measured in the morning after an overnight fast. Body composition was determined with the use of InBody 720 body composition analyzer (Biospace Corporation, Seoul, Republic of Korea). Body mass was recorded to the nearest value of 100 grams on calibrated digital scale.

### 2.6. Visceral Proteins

Serum albumin and prealbumin are frequently used laboratory parameters to measure nutritional status. In spite of their nonspecificity, they have been used to assess the change in nutritional status and stratifying risk of malnutrition [8].

### 2.7. Subjective Global Assessment (SGA)

subjective global assessment (SGA) is a simple evaluative tool that allows a physician to assess the patient's nutritional status [9]. Based on history taking and physical examination which is divided into 5 parts: weight change, dietary intake change, gastrointestinal symptoms, functional impairment, and physical examination (loss of subcutaneous fat, muscle wasting, edema). The results were obtained as normal (class A), suspected or moderate malnutrition (class B), and severe malnutrition (class C).

### 2.8. Dietary Assessment

Assessment of individual patient's oral intake was determined by the 3-day dietary recall method at baseline, the 4th week and the 8th week. 

### 2.9. Statistical Analysis

Baseline characteristics, blood chemistry test results, and body compositions of all subjects were reported by using mean ± standard deviation (SD). SGA was reported by frequency. Statistical analysis was conducted using SPSS software version 13.0 for windows. Means were compared by Wilcoxon signed-rank test and frequencies were compared by corrected chi-square test. *P* < 0.05 was considered statistically significant.

## 3. Results

The characterization of the study population is presented in Table 2. The sample is composed of 22 patients, 12 (54.5%) male and 10 (45.5%) female. The mean age of patients was 52.9 ± 12.8 years. The patient's cirrhosis had alcoholic etiology of 14 cases (63.6%). Fourteen (63.6%) patients had Child-Pugh A, 5 patients (22.7%) Child-Pugh B, and 3 patients (13.6%) Child-Pugh C cirrhosis. The mean body weight was 54.7 ± 1.9 kilogram. 

After vegetable protein supplementation, a significant improvement was demonstrated in the SGA class A from 10 patients (45.5%) to 16 (72.7%) and 18 (81.8%) at the 4th and 8th weeks, respectively (See Table 3). Patients in the SGA class B significantly decreased from 11 patients (50.0%) to 5 (22.7%) at the 4th and 8th weeks, respectively. At the 8th week, body weight and lean muscle mass were significantly increased from baseline 1.4 and 1.2 kilograms, respectively, which confirmed nutritional improvement in the patients. Moreover, BMI was increased from 21.4 to 21.9 kg/m^2^. No significant change in other nutritional parameters such as fat mass, Triceps skinfold thickness, albumin, and prealbumin was observed.

## 4. Discussions

Nutritional assessment is of crucial importance in the management of patients with liver cirrhosis. Malnutrition is common in liver cirrhosis and has an adverse effect on prognosis [10, 11]. Its early detection and treatment is thus of great clinical importance. This study showed that traditional nutritional status evaluation obtained by SGA was significant and early differences encountered between before and after vegetative protein supplementation. This improvement of nutritional status among these patients was confirmed by increased body weight, BMI, and lean muscle mass at the end of the study. 

Malnutrition has been found to be as common as 80% [12] among cirrhotic patients even in patients classified as Child-Pugh class A, the prevalence of malnutrition was as high as 25% [13]. Malnutrition is an independent risk factor for predicting survival in patients with cirrhosis. Short- and long-term studies have shown that nutritional supplements can improve survival in patients with cirrhosis. Those studies have suggested that recognition and treatment of malnutrition caused by cirrhosis are important. Body weight could lead to underestimated degree of malnutrition because of salt and water retention in cirrhotic patients. Body weight combined with lean muscle mass improves the validity of estimation for nutritional status in these patients. Although DEXA helps to improve nutritional assessment and follow up in cirrhotic patient, it is not available in many hospitals in Thailand. Anthropometric techniques may be affected by edema. Albumin and prealbumin are synthesized from liver; therefore, these are poor predictors for nutritional status in patients with cirrhosis [14]. SGA is a strong predictor of malnutrition in cirrhotic patients and it is applicable for use in clinical practice [15]. This study further supported the utility of the SGA in terms of followup for nutritional status in cirrhotic patient after vegetative protein supplementation.

## 5. Conclusions

Clinical assessment with the SGA in the form of single score and lean muscle mass are significant parameters to evaluate nutritional improvement in patients with cirrhosis after vegetative protein supplementation.

## Figures and Tables

**Table 1:** Nutritional content per pack of supplement.

	Vegetable protein (soy) supplementation
Energy (Kcal)	420
Protein (g)	20
Carbohydrate (g)	65
Fat (g)	10.6
Calcium (mg)	190
Sodium (meq)	72
Potassium (meq)	2400

**Table 2:** Characterization of the study population.

Variables	All patients
Sex (male/female)	12/10
Age (years)	52.9 ± 12.8
Child-Pugh—*n* (%)	
A	14 (63.6%)
B	5 (22.7%)
C	3 (13.6%)
Etiology—*n* (%)	
Alcohol	14 (63.6%)
Virus B	6 (27.3%)
Virus C	2 (9.0%)
Caloric intake (kcal/kg/day)	25.1 ± 5.6
Protein intake (g/kg/day)	1.1 ± 0.6

**Table 3:** Nutritional parameters in patients with cirrhosis at baseline and after vegetable protein supplementation^†^.

Variables	Baseline	4th week	8th week
Body weight (kg)	54.7 ± 1.9	55.4 ± 1.8	56.1 ± 1.8*
Body mass index (kg/m^2^)	21.4 ± 0.6	21.6 ± 0.7	21.9 ± 0.7*
Lean muscle mass (kg)	41.2 ± 3.3	41.7 ± 2.9	42.4 ± 2.7*
Fat mass (kg)	13.6 ± 0.9	13.5 ± 1.7	13.5 ± 1.4
Total body water (kg)	30.1 ± 2.2	30.5 ± 2.1	30.8 ± 1.9
Triceps skinfold thickness (mm)	10.9 ± 4.2	11.3 ± 3.5	11.4 ± 3.8
Albumin (g/dL)	3.8 ± 0.3	3.9 ± 0.2	3.9 ± 0.2
Prealbumin (g/dL)	1.4 ± 0.3	1.5 ± 0.3	1.5 ± 0.3
Subjective global assessment—*n* (%)			
A	10 (45.5%)	16 (72.7%)**	18 (81.8%)**
B	11 (50.0%)	5 (22.7%)**	4 (18.1%)**
C	1 (4.5%)	1 (4.5%)	0 (0%)

^†^Data are presented as mean ± SD.

^∗^ *P* < 0.05.

^∗∗^ *P* < 0.01.
